# The Characteristics of Heterozygous Protein Truncating Variants in the Human Genome

**DOI:** 10.1371/journal.pcbi.1004647

**Published:** 2015-12-07

**Authors:** István Bartha, Antonio Rausell, Paul J. McLaren, Pejman Mohammadi, Manuel Tardaguila, Nimisha Chaturvedi, Jacques Fellay, Amalio Telenti

**Affiliations:** 1 SIB Swiss Institute of Bioinformatics, Lausanne and Basel, Switzerland; 2 School of Life Sciences, École Polytechnique Fédérale de Lausanne, Lausanne, Switzerland; 3 Vital-IT group, SIB Swiss Institute of Bioinformatics, Lausanne, Switzerland; 4 Computational Biology Group, ETH Zurich, Zurich, Switzerland; 5 J. Craig Venter Institute, La Jolla, California, United States of America; Pierre and Marie Curie University (UPMC), FRANCE

## Abstract

Sequencing projects have identified large numbers of rare stop-gain and frameshift variants in the human genome. As most of these are observed in the heterozygous state, they test a gene’s tolerance to haploinsufficiency and dominant loss of function. We analyzed the distribution of truncating variants across 16,260 autosomal protein coding genes in 11,546 individuals. We observed 39,893 truncating variants affecting 12,062 genes, which significantly differed from an expectation of 12,916 genes under a model of neutral *de novo* mutation (*p*<10^−4^). Extrapolating this to increasing numbers of sequenced individuals, we estimate that 10.8% of human genes do not tolerate heterozygous truncating variants. An additional 10 to 15% of truncated genes may be rescued by incomplete penetrance or compensatory mutations, or because the truncating variants are of limited functional impact. The study of protein truncating variants delineates the essential genome and, more generally, identifies rare heterozygous variants as an unexplored source of diversity of phenotypic traits and diseases.

## Author Summary

Genome sequencing provides evidence for large numbers of putative protein truncating variants in humans. Most truncating variants are only observed in few individuals but are collectively prevalent and widely distributed across the coding genome. Most of the truncating variants are so rare that they are only observed in heterozygosis. The current study identifies 10% of genes where heterozygous truncations are not observed and describes their biological characteristics. In addition, for genes where rare truncations are observed, we argue that these are an unexplored source of diversity of phenotypic traits and diseases.

## Introduction

Recent population expansion and limited purifying selection have led to an abundance of rare human genetic variation [1–3] including stop-gain and frameshift mutations. Thus, there is increasing interest in the identification of natural human knockouts [3–8] through the cataloguing of homozygous truncations. However, heterozygous truncation can also lead to deleterious functional consequences through haploinsufficiency due to decreased gene dosage, or through a dominant-negative effect [9,10]. In order to quantify the importance of heterozygous protein truncating variation, we characterized genes showing fewer *de novo* truncations in the general population than expected under a neutral model. We hypothesized that there is a set of genes that cannot tolerate heterozygous protein truncating variants (PTVs) because of early life lethality.

## Results

### Fewer genes carry heterozygous PTVs than expected under neutral evolution

We used stop-gain (nonsense) single nucleotide variants and frameshift (insertions/deletions) variants to assess tolerance to heterozygous PTVs across the human genome. We considered transcripts from 16,260 autosomal protein coding genes annotated by the consensus coding sequence (CCDS) project [11], for which *de novo* mutation rate estimates were recently calculated [12], and where the number of synonymous variants in sequenced individuals followed expectation (**Methods**). The study dataset included 11,546 exomes in which we observed 39,893 rare PTVs (allele frequency < 1%), affecting 12,062 (74.1%) genes.

**Fig 1:**
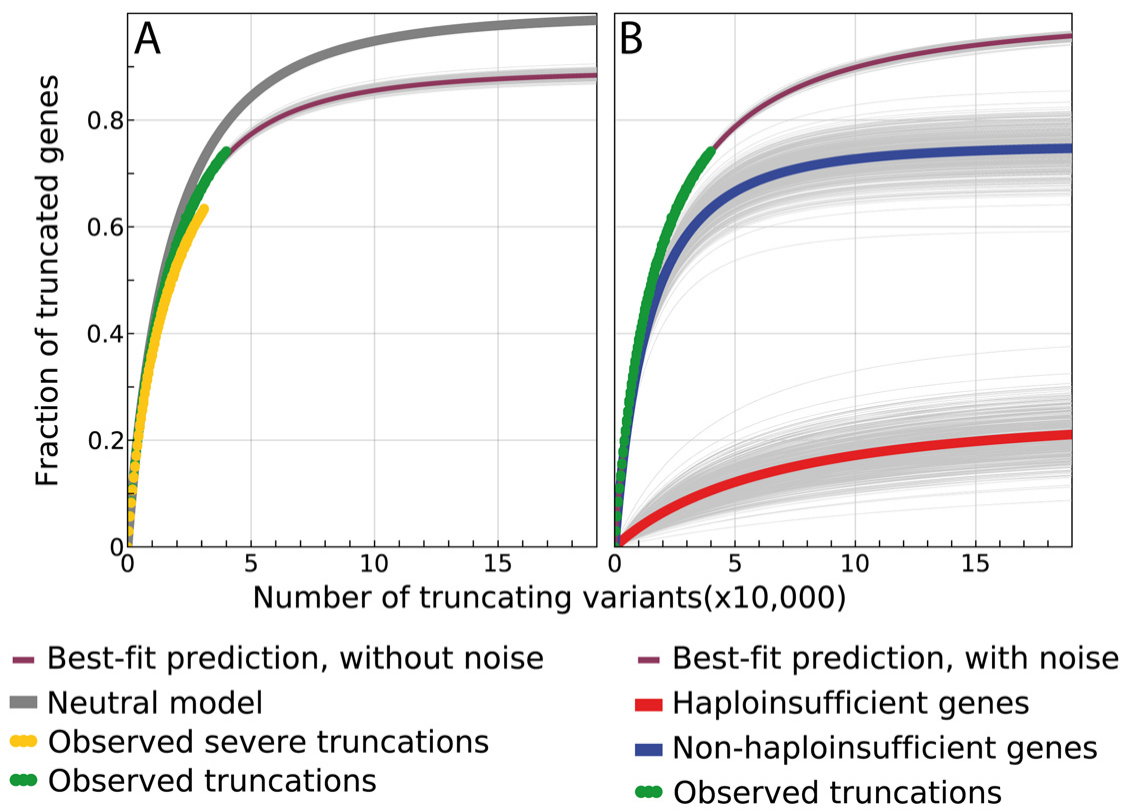
Observed and expected PTVs in the study population. **A**: Fraction of genes with at least one stop-gain or frameshift variant as a function of the number of sampled PTVs. The gray curve shows the expected number of genes under a model of neutral de novo mutation rate [12] representing the null hypothesis (no deleterious effects). The green curve shows the number of genes observed with at least one PTV. The orange curve limits the number of observed genes to those hosting highly damaging variants [13]. The purple curve shows the predicted number of genes with at least one PTV under the estimated best-fit parameters under model A–bootstrap replicas of this fit is shown by pale gray (see Methods). **B**: Extrapolation of the observed number of genes with at least one PTV assuming a model that includes the possibility of finding PTVs due to biological and technical noise. The purple curve shows the predicted number of genes with at least one PTV under the estimated best-fit parameters, while the green curve shows the observed data. Decomposition of the observed and predicted number of genes with at least one PTV: variants in non-haploinsufficient genes (blue) saturate early; variants found in haploinsufficient genes (red) continue to accumulate PTVs due to the constant contribution of biological and technical noise.

To test whether there is a subset of genes that are intolerant to heterozygous truncation, we simulated a model of generation of neutral *de novo* PTVs for all genes (i.e. assuming viability of affected individuals). By randomly assigning 39,893 hypothetical stop-gain and frameshift variants to genes according to their *de novo* mutation rate [12], we observed that 12,916 out of 16,260 genes (95% CI, 12,805–12,991) would be expected to carry at least one stop-gain or frameshift variant. The expected number of genes is significantly greater than the 12,062 truncated genes observed in the study dataset for the same number of PTVs (6.6% depletion, empirical p-value computed by Monte Carlo simulation < 10^-4^; **Fig 1A**). The depletion in number of observed truncated genes was greater when severe PTVs, i.e. those predicted to have the greatest functional impact [13], were considered (n = 10,340 vs. a neutral expectation of 11,821–11,978; 13.1% depletion p < 10^-4^). This suggests that a measurable fraction of *de novo* heterozygous stop-gain and frameshift variants are highly deleterious and hence under strong purifying selection. Hereafter we denote that fraction as the haploinsufficient genome (*f*_*hi*_).

### Characteristics of genes comprising the haploinsufficient genome

We assessed the functional properties of the subset of genes that were not observed to carry PTVs (n = 4,198) (**Table 1)**. These genes were highly conserved, had fewer paralogs, were more likely to be part of protein complexes and were more connected in protein-protein interaction networks than the rest of the genes. Furthermore, they had characteristics of essentiality and haploinsufficiency, and a higher probability of CRISPR-Cas9 editing compromising cell viability [14]. The set of genes not carrying PTVs was enriched in OMIM genes annotated with ‘haploinsufficient’ or ‘dominant negative’ keywords [15], and was enriched in genes associated with increased mortality in mouse models [16] (**Table 1**). Non truncated genes were overrepresented in functional categories such as transcription regulation, developmental processes, cell cycle, and nucleic acid metabolism (**S1 Table)**, in line with earlier characterization of haploinsufficient genes [17]. Together, these results indicate that a number of basic cellular functions depend on the integrity of coding and expression of both alleles of component genes. The enrichment pattern was the opposite for the set of 2347 genes with homozygous PTVs. In particular genes with homozygous PTVs have more paralogs, are less likely to be part of protein complexes, have a smaller posterior probability of haploinsufficiency, are depleted in genes which affect cell viability in CRISPR-Cas9, have higher dN/dS values, are less likely to be essential, have lower connectivity indices, are depleted in ClinVar and OMIM, and are depleted in genes associated with increased mortality in mice. All these observations are significant and details are listed in **S4 Table**.

**Table 1:** Characteristics of the subset of genes (n = 4,204) observed without PTVs after sequencing 16,260 protein coding autosomal genes in 11,546 individuals. Tests compare genes with and without heterozygous PTVs.

Annotation	Effect in non-truncated genes	P-value	Test	Data Source
dN/dS	Lower (conservation)	1E-295	Rank-sum test	Ensembl primate genomes[13]
Paralog count	Lower	4E-94	Poisson regression	Ensembl Biomart
Loss of cell viability (CRISPR-Cas9)	Enrichment	3E-16	Logistic regression	Shalem et al. 2014 [14]
Part of a protein complex	Enrichment	3E-29	Logistic regression	Gene Ontology term “Protein complex” GO:0043234
Essentiality	Higher	4E-34	Logistic regression	OGEE (http://ogeedb.embl.de/)
Connectivity in protein-protein interaction network	Higher	5E-52	Linear regression	OGEE (http://ogeedb.embl.de/)
Predicted haploinsufficiency	Higher	1E-162	Linear regression	Huang et al. 2010 [10]
OMIM ‘haploinsufficient’ and ‘dominant negative’ subset	Enrichment	5E-12	Logistic regression	Petrovski et al. 2013[15]
Mouse knock-out mortality phenotype	Enrichment	5E-63	Logistic regression	Mouse/Human Orthology with Phenotype Annotations [16]

### Estimating the fraction of genes intolerant to heterozygous stop-gain and frameshift variants

Genes without PTVs in our analysis may be truly part of the haploinsufficient genome or the result of insufficient sample size to detect rare events. Thus, we next sought to estimate the total haploinsufficient fraction (*f*_*hi*_) of the genome in the full population by a modeling approach. Assuming that a fraction *f*_*hi*_ of genes do not carry *de novo* PTVs while the remaining genes do so according to their neutral mutation rates [12], *f*_*hi*_ can be estimated by fitting a model to the observed relative distribution of PTVs (relative to the rest of genes; **Methods**). This analysis estimates a fraction of the haploinsufficient genome of *f*_*hi*_ *=* 10.8% (95% CI = 9.5–11.7%) of protein coding genes (**Fig 1A**).

Some genes may tolerate PTVs because their functional effects are masked by incomplete penetrance [18], by compensatory variants [19], or because of a low functional impact of the truncation [13]. In addition, false positive errors in sequencing and variant calling procedures contribute to the distribution of observed variants [20–22]. We collectively treated these factors as noise, because they can lead to the observation of a truncated gene in a viable individual without truly probing the general viability of carrying only one functional allele in a given gene. Therefore, we extended our model to allow for the possibility of observing PTVs in the haploinsufficient fraction of the genome by introducing a second parameter representing the number of variants originating either from biological (incomplete penetrance, compensatory variants and low impact truncation) or technical noise (false positive sequencing or variant calling errors) (**Methods,** model A). Using this extension, the estimated fraction of genes intolerant to PTVs increased to 24.4% (95% CI, 18.3–32.1%, **Fig 1B**).

An important consequence of biological and technical noise is that the fraction of genes bearing PTVs does not saturate as a function of the number of observed PTVs, but keeps rising. Our model predicts that after having sequenced 40,000 exomes (representing a sample of approximately 90,000 PTVs) more than 50% of newly identified truncated genes will result from biological and technical noise (**S1 Fig)**—an important consideration for ongoing sequencing programs and interpretation of resources, such as that of the Exome Aggregation Consortium (ExAC, http://exac.broadinstitute.org). At the sample size of 40,000 exomes, and with 2 to 6% of all observed truncations due to technical errors [5,6,8], 400 to 1025 genes intolerant to PTVs will exhibit truncations due to sequencing and variant calling errors. For the same sample size, 2345 to 2549 genes intolerant to PTVs will exhibit truncations due to incomplete penetrance, compensatory variants or low impact truncation.

We next assessed the robustness of these estimates using an alternative approach that models the expected number of PTVs as a function of the observed synonymous coding variants (**Methods,** model B). This model assumes that, in the absence of deleterious consequences, the number of heterozygous PTVs correlates with the number of synonymous variants observed in a gene. This approach resulted in highly similar estimates of *f*_*hi*_ (26.1%, 95% CI 19.7–34.1%) compared to the previous model. The posterior probabilities from model B are highly correlated with two published scores for haploinsufficiency[10,23] (Spearman R > 0.31, p-value<2.2e-16 in both cases). All three approaches showed a similar predictive power for 175 known haploinsufficient genes causing Mendelian disorders[15] (**S2 Fig**).

**Fig 2:**
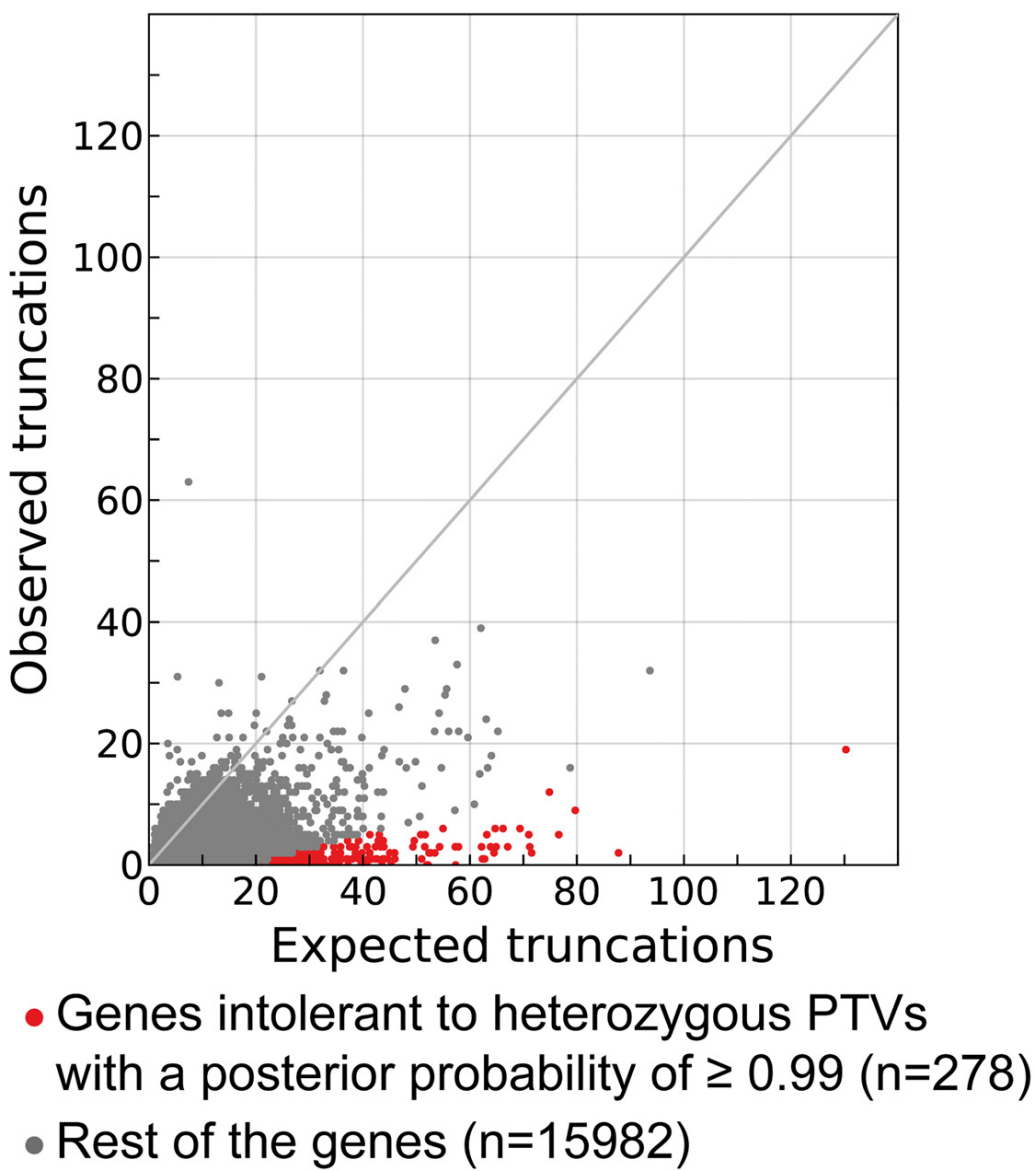
Expected and observed number of PTVs per gene. Each dot in the scatter plot corresponds to a gene. X-axis reflects the expected number of PTVs for each gene according to a model of neutral variation based on synonymous variants (Model B, see Methods) while on Y-axis indicates observed number of PTVs in the study dataset. Genes intolerant to heterozygous PTVs with a posterior probability of ≥ 0.99 are colored in red. The distribution shows that there is a continuum of intolerance to PTVs with a general paucity of observed versus expected truncations in the coding genome. The gray line has a slope of 1.

Model B also underscores that there is a continuum of tolerance to heterozygous truncation, with a large number of genes harboring fewer heterozygous PTVs than expected under a neutral model (**Fig 2**). It is however important to indicate that long genes have a high number of expected PTVs, thus the observation of a small number of PTVs in these genes still reflects a strong depletion and high posterior probability of being intolerant to heterozygous truncations. Indeed, of the 282 genes with a posterior probability of being intolerant to heterozygous truncation higher than 0.99, 155 have observed PTVs (**Fig 2 and S2 Table**). As expected, genes highly depleted of PTVs show similar properties to the genes without any PTVs (**S3 Table**). In particular, they are enriched for known haploinsufficient genes associated with Mendelian diseases. The comparison between the observed and expected number of PTVs in a gene is key to evaluating its functional tolerance to truncation.

## Discussion

This work identifies a substantial proportion of genes that do not tolerate loss of one of the two gene copies, and by the evidence for a gradient of haploinsufficiency across a large proportion of the coding genome. Heterozygous PTVs are rarely compensated at the gene expression level, as shown in our previous work [13] and in recent analyses [7]. Despite the absence of dosage compensation, Rivas et al. suggest that homeostatic mechanisms at the cellular level maintain biological function [7]. However, we show clear evidence that over 10% of the genes cannot be compensated, while an additional 10 to 15% of truncated genes may be rescued by incomplete penetrance or compensatory mutations, or because the truncating variants are of limited functional impact.

The importance of these variants has also been observed in model organisms. Studies in mice show that when homozygous knockout mutants are not viable, up to 71.7% of heterozygous PTVs have phenotypic consequences [24]. The systematic phenotyping of knockout mice also demonstrates that haploinsufficiency might be more common than generally suspected [25]. However, a practical limitation of the above approaches, in particular in animal studies, is that observation of phenotypes resulting from damaging mutations may require exposure to specific triggers or environmental interactions [6,25]. In contrast, in humans, life-long exposures may eventually reveal a phenotypic trait or disease associated with heterozygous gene truncations [8]. Here, clinical symptoms could be observed later in life, and present sporadically–not necessarily within a pedigree. This is illustrated by a recent report on the consequences of haploinsufficiency of cytotoxic T-lymphocyte-associated protein 4 gene (*CTLA-4*) presenting as undiagnosed or misdiagnosed sporadic autoimmune disorder in the second to fifth decades of life [26]. Despite the prevalence of rare heterozygous PTVs, there has been more attention to the occurrence of homozygous truncations (human knockouts). However, the genes that are observed with biallelic PTVs have, as a set, characteristics of dispensability: less conservation, greater redundancy, less biological and cellular centrality, and limited essentiality in mice and cellular models. Thus, we argue that homozygous truncations result from high allele frequency variants that are less likely to carry functional consequences (the exception being recessive disorders in a population).

There are a number of possible limitations to the present study. In the modeling work, we analyzed rare variants (less than <1% allele frequency) to focus on *de novo* events and for consistency with the *de novo* mutation rates estimated by Samocha et al. [12]. Nevertheless, our estimates held true when the whole analysis was repeated with the smaller subset of singleton variants—singletons possibly reflect false positive sequencing and alignment calls. It was also repeated with all variants irrespective of allele frequency (instead of analyzing variants of less than 1% allele frequency) (**S3 Fig**). Initially we omitted splice-site variants because of less predictability of the functional consequences. Extending the analysis to include splice-site variants did not change the results and conclusions (**S3 Fig** and **S5 Table**). This demonstrates that the results do not originate from a specific subset of variants. We did not have primary control on sequencing coverage for some of the exome sequence datasets that could result in ascertainment errors. To correct for this potential bias, we discarded genes where the observed number of synonymous mutations deviated from expectation. The intolerance of genes to *de novo* truncation was assessed across combined human populations. Therefore, estimations of the haploinsufficient genome account for the fraction of haploinsufficient genes common to all humans. Intolerance to heterozygous PTVs should be regarded as a different concept than gene sequence conservation. PTVs in a conserved gene might have a recessive mode of inheritance and are thus potentially observable in a viable individual. On the other extreme, positively selected genes could be haploinsufficient upon heterozygous truncation. These considerations notwithstanding, we consistently identified a quantifiable fraction of the human genome that is intolerant to heterozygous PTVs, with an estimated lower bound of 9.5%.

The prevalent nature of rare heterozygous PTVs suggests that a map of “essentiality” on the basis of dominant loss of function is within reach. The concept of the essential genome has been explored in analyses of minimal bacterial genomes [27], mouse knockout studies [28], studies of transposon or chemical mutagenesis [29], and in studies that used CRISPR-Cas9 genome-editing technology [14,30]. Here, we propose that mapping the haploinsufficient genome will improve the understanding of the genetic architecture of diseases. In agreement with the recent work of Li et al.,[6] we argue that the burden of rare human heterozygous variation is an unexplored source of diversity of phenotypic traits and diseases.

## Methods

### Exomes

We collected exome data from public and non-public sources [31–38] (**S6 Table**). With the exception of the Swiss HIV Cohort Study these sources are not disease-specific cohorts. Variants were filtered based on Hardy-Weinberg equilibrium (discarded if p <1x10^-8^). For public data sets, variants were called at the data source with their respective pipelines. For non-public data sets, sequence reads were aligned using BWA, and called with Haplotypecaller using GATK 3.1. Variants were annotated with SnpEff 3.1 and filtered as described in [39–41]. Only transcripts from autosomal protein coding genes reliably annotated by the Consensus Coding Sequence (CCDS, Release 12 04/40/2013) project[11] that underwent the full process of CCDS curation ('Public' status in CCDS terminology, n = 17,756) were considered. As a reference background throughout all analyses, a total number of 16,521 autosomal protein coding genes was obtained by considering genes with available *de novo* mutation rate from Samocha et al. [12] and with at least one synonymous, missense, stop-gain or frameshift variant detected in the exome data. We discarded genes where the observed number of synonymous mutations deviated from expectation (see below). For consistency with [12], we only retained variants mapping within the limits of the reference transcript used to assess the *de novo* mutation rate per gene. Furthermore, only rare stop-gain and frameshift variants (allele frequency <1%) were considered to assess the deviation from neutral expectations. Throughout the study we considered each rare variant as a single *de novo* event of mutation, irrespective of the number of individuals in which it was observed.

### Models of haploinsufficiency and noise

Under a neutral model, the expected number of *de novo* PTVs (stop-gain or frameshift) in a gene is determined by its probability of *de novo* mutation (assessed from the sequence context and gene length) [12] and the number of sequenced individuals. However, potential intolerance to heterozygous truncation would decrease the expected number of *de novo* PTVs as a consequence of embryonic or early life lethality. To model the expected number of variants in a gene accounting for potential deleterious effects, we used two approaches.

First we evaluated the relative distribution of PTVs across genes (hereafter the model A). This model assumes that genes tolerating heterozygous truncation will be found truncated in the population according to their relative probability of *de novo* mutation (relative to the rest of genes), while a fraction of genes will not be observed as truncated due to early lethality. Alternatively, we assessed a second model (hereafter the model B) in which the absolute number of *de novo* PTVs in a gene is estimated from the probability of *de novo* PTVs and the absolute number of observed *de novo* synonymous coding variants in that gene.

**Model A** is formulated as follows. The total number *V* of observed PTVs is composed of a fraction *V e* of false positive variants (including sequencing errors and incomplete penetrance) and the complementary fraction *V*(1 − *e*). We assume that the total set of genes G can be divided in two classes of genes, named HI for the haploinsufficient class and HS for non-haploinsufficient class of relative sizes *f*_*hi*_ and (1 − *f*_*hi*_ *)* respectively. We assume that the fraction of variants *V e* is distributed across all genes proportionally to their relative de novo neutral mutation rates. However, the *V*(1 − *e*) fraction of variants should only be observed in the (1 − *f*_*hi*_ *)* fraction of HS genes. Therefore, in model A the expected number of variants in a HI gene *g* is

$$E_{g|HI}^{trunc}\;=V\;e\frac{p_{g}^{trunc}}{\sum_{i\in G}\;p_{i}^{trunc}}$$

while the expected number of variants in a HS gene *g* is

$$E_{g|HS}^{trunc}\;=V\;e\frac{p_{g}^{trunc}}{\sum_{i\in G}\;p_{i}^{trunc}}\;+V(1-e)\frac{p_{g}^{trunc}}{\sum_{i\in HS}\;p_{i}^{trunc}}$$

Assuming that $1-f_{hi}\;=\frac{\sum_{i\in HS}\;p_{i}^{trunc}}{\sum_{i\in G}\;p_{i}^{trunc}}$, then

$$E_{g|HS}^{trunc}\;=\;V\;e\frac{p_{g}^{trunc}}{\sum_{i\in G}\;p_{i}^{trunc}}\;+V(1-e)\frac{p_{g}^{trunc}}{\sum_{i\in G}\;p_{i}^{trunc}}\frac{1}{1-f_{hi}}$$

We note that model A is based on observed variants and therefore false negative errors are not considered.

To formulate **model B**, we assume that the expected number of *de novo* synonymous mutations in a gene *g* is

$$E_{g}^{syn}\;=M\;p_{g}^{syn},$$

where $p_{g}^{syn}$ is the *de novo* rate of synonymous mutations in a gene *g* and *M* is a constant. Following [12] we estimate *M* from the regression of the observed number of synonymous mutations ($O_{g}^{syn})$ in a gene on $p_{g}^{syn}$:

$$O_{g}^{syn}\;=M\;p_{g}^{syn}+e.$$

To avoid genes with low coverage, we disregarded from the analysis those genes whose residual in the above regression is higher than 3 times the standard deviation of all residuals. We note that, in contrast to [12] we omit the intercept term in this regression, because we expect no variants in a gene for which $p_{g}^{syn}$ equals zero.

Having estimated *M*, the expected number of PTVs in a gene *g* is given by:

$$E_{g}^{trunc}\;=\;Mp_{g}^{trunc}.$$

Introducing gene specific deviations from the neutral expectation as well as for systematic errors, the number of observed PTVs can be written as:

$$E_{g}^{trunc}\;=\;M\;p_{g}^{trunc}\;s_{g},$$

where *s*_*g*_ accounts for gene specific differences. We do not estimate *s*_*g*_ for each gene, but assume that genes can be classified into two groups (haploinsufficient and non-haploinsufficient), each having a distinct class specific value (*s*_*HI*_ and *s*_*HS*_):

$$E_{g|HS}^{trunc}\;=\;M\;p_{g}^{trunc}\;s_{HS}$$

$$E_{g|HI}^{trunc}\;=\;M\;p_{g}^{trunc}\;s_{HI}$$

*s*_*HI*_ and *s*_*HS*_ include the sum effect of systematic false positive and negative errors, as well as class specific differences in the penetrance mutations, however it is not possible to separate these individual components.

Both in model A, or in model B, to estimate the fraction of genes intolerant to heterozygous PTVs we use the following mixture model. We define a random variable *x*_*g*_ as the number of PTVs in gene *g*. A latent random variable *z*_*g*_ can take two values: *HI* or *HS* and has the probability density distribution:

$$P\left(z_{g}\;=\;HI\right):=\;f_{hi}$$

$$P\left(z_{g}\;=\;HS\right):=\;1-f_{hi}$$

where the parameter *f*_*hi*_ represents the fraction of genes intolerant to heterozygous PTVs. The conditional probability distribution of *x*_*g*_ given *z*_*g*_ is defined as:

$$P\left(x_{g}\;=\;k|z_{g}\;=\;HI\right)\;=\;\text{Poisson}\left(k,\lambda_{HI}\right)$$

$$P\left(x_{g}\;=\;k|z_{g}\;=\;HS\right)\;=\;\text{Poisson}\left(k,\lambda_{HS}\right)$$

$$\;\lambda_{HS}\;=\;E_{g|HS}^{trunc}$$

$$\;\lambda_{HI}\;=\;E_{g|HI}^{trunc}.$$

where $E_{g|HS}^{trunc}$ and $E_{g|HI}^{trunc}$ are the expected number of PTVs in a gene g from the HS and the HI classes respectively as formulated in either model A or model B. Marginalizing over the values of the latent variable z_g_ yields the probability density distribution of *x*_*g*_ as:

$$P\left(x_{g}=k\;\right)=f_{hi}\;\text{Poisson}\left(k,\lambda_{HI}\right)+\left(1-f_{hi}\right)\;\text{Poisson}\left(k,\lambda_{HS}\right).$$

The probability that any gene acquires *k* variants is:

$$P\left(X=k\right)=\frac{\sum_{g}P\left(x_{g}=k\;\right)}{n_{genes}},\;\text{where}\;n_{genes}\;\text{is the total number of genes}.$$

The model’s parameters (*e* and *f*_*hi*_ in model A, and *f*_*hi*_, *s*_*HI*_ and *s*_*HS*_ in model B) are estimated by fitting the cumulative density distribution of *X* to the empirical cumulative density distribution of the data by least-squares fitting using the Nelder-Mead simplex numerical optimization algorithm (as implemented in the Apache Commons Math library). This method provided better estimates for reproducing the distribution of variant counts per gene compared to other alternatives considered (**S4 Fig**). In order to estimate the variability of the inferred model parameters we repeated the parameter estimation on 500 bootstrap replicates. Each bootstrap replicate was generated by resampling the list of genes with replacement.

Using the estimated parameters we calculate the posterior probability of haploinsufficiency for gene *g* as:

$$P\left(z_{g}=HI|x_{g}=o_{g}\right)=\frac{P\left(z_{g}=HI\right)P\left(x_{g}=o_{g}\;|z_{g}=HI\right)}{P\left(x_{g}=o_{g}\right)},$$

where *o*_*g*_ is the observed number of PTVs in the gene *g*.

### Characteristics of haploinsufficient genes

Gene sets were obtained from the Reactome pathway database version 40 (http://www.reactome.org/). dN/dS values were assessed as described in [13]. Degree of connectivity in the protein-protein interaction network was obtained from the OGEE database (http://ogeedb.embl.de/). Paralogs were counted using Ensembl Biomart’s 'Human Paralog Ensembl Gene ID' attribute. Genes in protein complexes were obtained from Gene Ontology term GO:0043234 (named “protein complex”). Genes affecting cell viability in CRISPR-Cas9 experiments were collected from [14,30]. Severity of protein truncation was assessed by the NutVar score (http://nutvar.labtelenti.org) [13]. Phenotypic consequences in mouse models were downloaded from ftp://ftp.informatics.jax.org/pub/reports/HMD_HumanPhenotype.rpt and filtered for the Mammalian Phenotype Ontology term “Mortality/Aging” (MP:0010768, MP:0005374, MP:0005373, MP:0005372). For the assessment of depletion or enrichment of functional gene sets we used one tailed hypergeometric test. We adjusted the p-values by the Benjamini- Hochberg method to correct for multiple testing. We tested pathways with at least 100 elements only.

We estimated the number of exomes required for a certain number of sampled PTVs using the jackknife projection as in [42].

## Supporting Information

S1 Table Enrichment tests results against Reactome pathways for genes without PTVs. Only significant results are shown as judged by 5% FDR calculated using the Benjamini-Hochberg procedure.
(XLSX)

S2 Table List of analyzed genes with their number of observed PTVs, expected PTVs and posterior probability of being intolerant to heterozygous PTVs. (TXT)

S3 Table Characteristics of the subset of genes (n = 282) having a higher than 0.99 probability of being intolerant to heterozygous truncation. Tests compare genes with posterior probability > 0.99 vs the rest of the genes.
(XLSX)

S4 Table Characteristics of the subset of genes (n = 2347) having at least one homozygous PTV. Tests compare genes with at least one homozygous PTV vs the rest of the genes.
(XLSX)

S5 Table Characteristics of the subset of genes having no PTV, even if splice-site variants are also considered. Tests compare genes without PTV vs the rest of the genes. Attached as separate file.
(XLSX)

S6 Table Data sources. (XLSX)

S1 Fig Conditional probability that when observing a gene truncated for the first time, the gene is intolerant to PTVs. When the conditional probability crosses 50% (at 90,000 PTVs) biological and technical noise become the main source of truncations. We estimate that 40,000 exomes are required to sample 90,000 PTVs using the jackknife projection as in [42].
(PDF)

S2 Fig Predictive power for annotated haploinsufficient genes. The figure shows the receiver operating characteristic (ROC) curves of the classification power of the gene posterior probabilities of being haploinsufficient assessed in this work (black), the haploinsufficiency score from Huang et al 2010 (blue) and the GHIS score from Steinberg et al. 2015 (green) for a lists of 175 OMIM haploinsufficient genes as determined by Petrovski et al 2013. AUC values are: 0.762 (this work), 0.780 (Huang et al), 0.694 (GHIS). We used the rest of the genes as the negative set. We note that both Huang et al 2010 and Steinberg et al. 2015 included in their learning set 94 genes in common with the OMIM haploinsufficient genes used here.
(PDF)

S3 Fig Distribution of parameter estimates and predictions of the model A. Analysis considers only singletons (A-C), all variants irrespective of allele frequency (D-F) or rare variants (G-I).
(PNG)

S4 Fig Distribution of variant counts per gene as observed or predicted under best-fit parameters of model A using 3 different estimation techniques. **A:** linear space, **B:** log space. Black curve: observed counts, red curve: prediction based on least-squares fit to the cumulative distribution function (see Methods), green curve: maximum likelihood estimate, blue curve: least squares fit to the accumulation curve of truncated genes as shown in Fig 1. The CDF method was chosen and maximum likelihood was discarded because its estimates did not fit the observations.
(PDF)
