# Supplementary material for: The Characteristics of Heterozygous Protein Truncating Variants in the Human Genome
Source: PLoS Comput Biol. 2015 Dec 7;11(12):e1004647. doi: 10.1371/journal.pcbi.1004647 (PMC4671652; doi:10.1371/journal.pcbi.1004647)
Supplement: S1 Fig — When the conditional probability crosses 50% (at 90,000 PTVs) biological and technical noise become the main source of truncations. We estimate that 40,000 exomes are required to sample 90,000 PTVs using the jackknife projection as in [42]. (PDF) [file pcbi.1004647.s007.pdf]

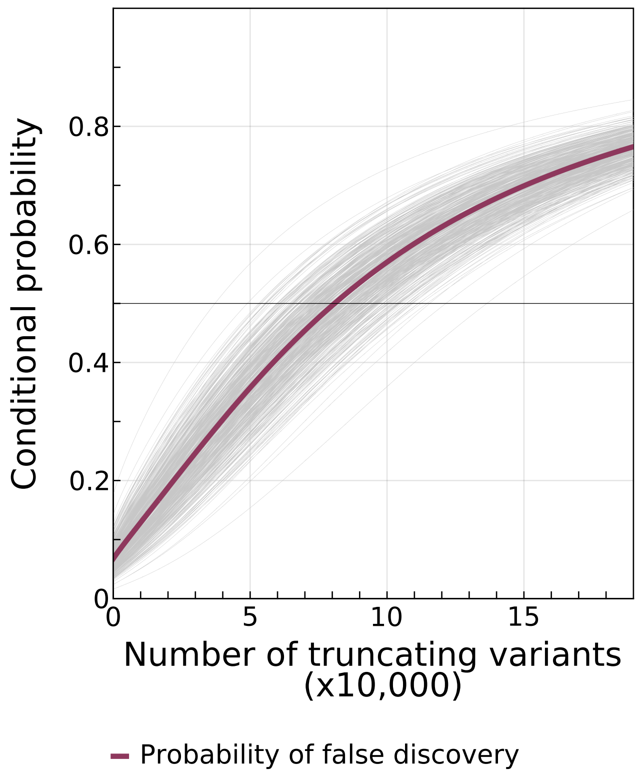

Figure S1: **Conditional probability that when observing a gene truncated for the first time, the gene is intolerant to PTVs.** When the conditional probability crosses 50% (at 90,000 PTVs) biological and technical noise become the main source of truncations. We estimate that 40,000 exomes are required to sample 90,000 PTVs using the jackknife projection as in[42].
