# Supplementary material for: The Characteristics of Heterozygous Protein Truncating Variants in the Human Genome
Source: PLoS Comput Biol. 2015 Dec 7;11(12):e1004647. doi: 10.1371/journal.pcbi.1004647 (PMC4671652; doi:10.1371/journal.pcbi.1004647)
Supplement: S2 Fig — The figure shows the receiver operating characteristic (ROC) curves of the classification power of the gene posterior probabilities of being haploinsufficient assessed in this work (black), the haploinsufficiency score from Huang et al 2010 (blue) and the GHIS score from Steinberg et al. 2015 (green) for a lists of 175 OMIM haploinsufficient genes as determined by Petrovski et al 2013. AUC values are: 0.762 (this work), 0.780 (Huang et al), 0.694 (GHIS). We used the rest of the genes as the negative set. We note that both Huang et al 2010 and Steinberg et al. 2015 included in their learning set 94 genes in common with the OMIM haploinsufficient genes used here. (PDF) [file pcbi.1004647.s008.pdf]

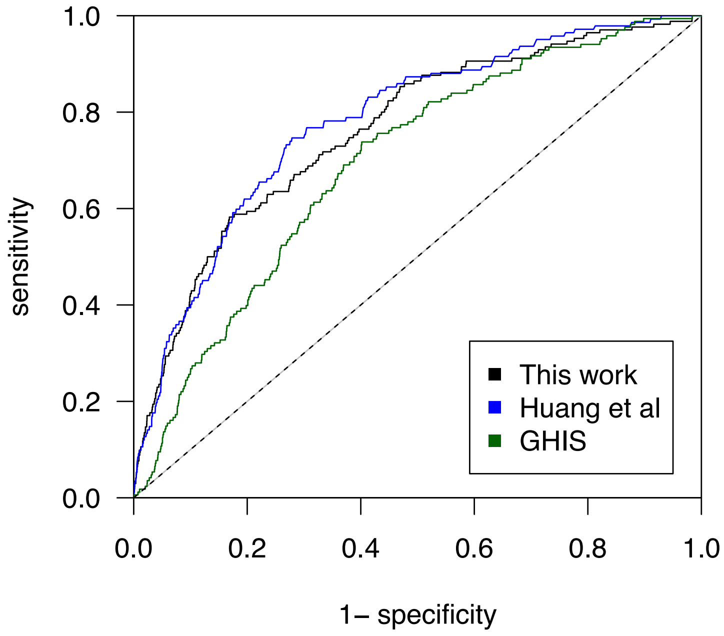

Figure S2: Predictive power against annotated haploinsufficient genes of the posterior probability of being intolerant to heterozygous truncation. The figure shows the receiver operating characteristic (ROC) curves of the classification power of the gene posterior probabilities of being haploinsufficient assessed in this work (black), the haploinsufficiency score from Huang et al 2010 (blue) and the GHIS score from Steinberg et al. NAR 2015 (green) for a lists of 175 OMIM haploinsufficient genes as determined by Petrovski et al 2013. AUC values are: 0.762 (this work), 0.780 (Huang et al), 0.694 (GHIS). We used the rest of the genes as the negative set. We note that both Huang et al 2010 and Steinberg et al. NAR 2015 included in their learning set 94 genes in common with the OMIM haploinsufficient genes used here.
