# Supplementary figures and images for: The Characteristics of Heterozygous Protein Truncating Variants in the Human Genome
Source: PLoS Comput Biol. 2015 Dec 7;11(12):e1004647. doi: 10.1371/journal.pcbi.1004647 (PMC4671652; doi:10.1371/journal.pcbi.1004647)

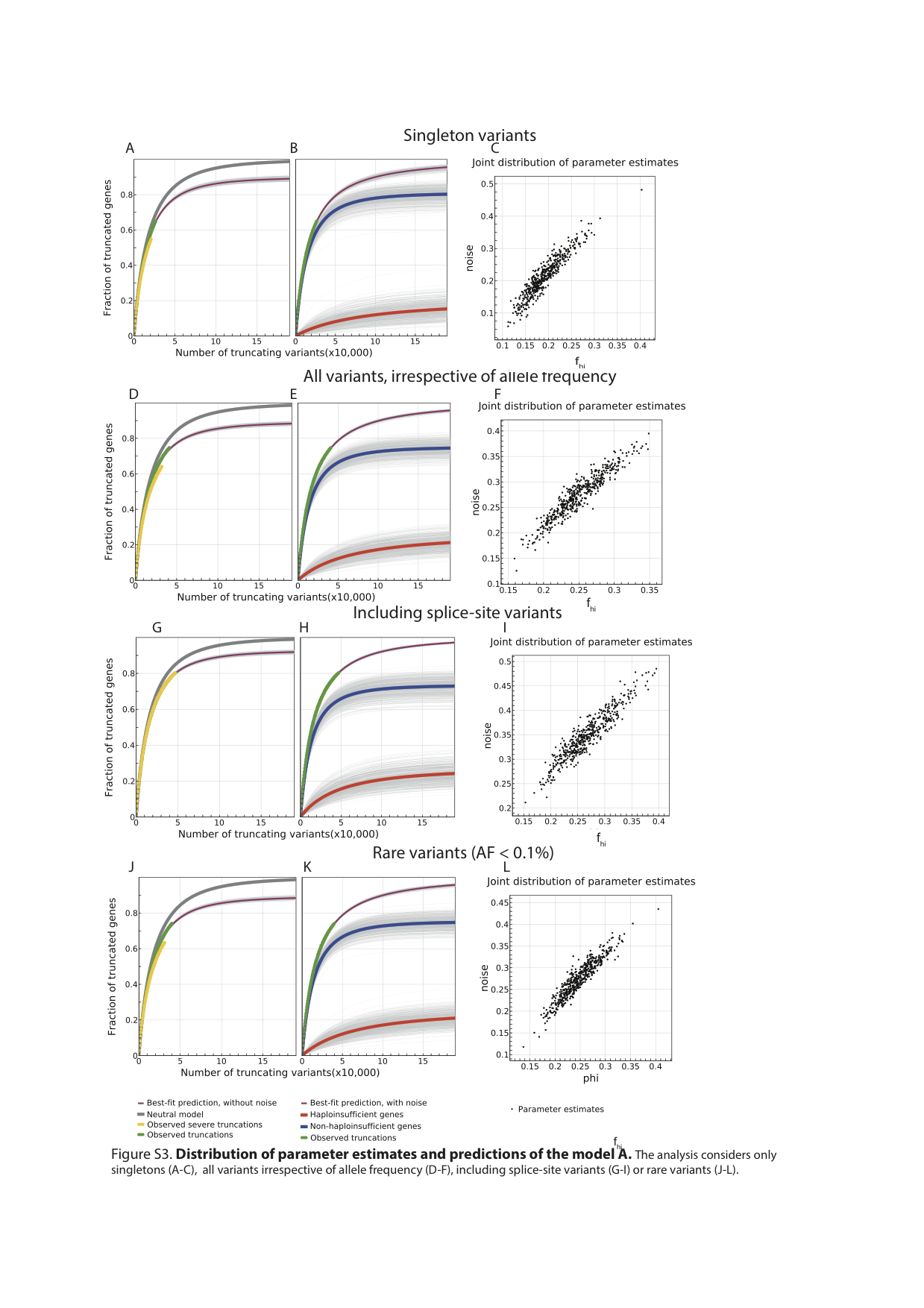

Supplement: S3 Fig — Analysis considers only singletons (A-C), all variants irrespective of allele frequency (D-F) or rare variants (G-I). (PNG) [file pcbi.1004647.s009.png]
