# Supplementary material for: The Characteristics of Heterozygous Protein Truncating Variants in the Human Genome
Source: PLoS Comput Biol. 2015 Dec 7;11(12):e1004647. doi: 10.1371/journal.pcbi.1004647 (PMC4671652; doi:10.1371/journal.pcbi.1004647)
Supplement: S4 Fig — A: linear space, B: log space. Black curve: observed counts, red curve: prediction based on least-squares fit to the cumulative distribution function (see Methods), green curve: maximum likelihood estimate, blue curve: least squares fit to the accumulation curve of truncated genes as shown in Fig 1. The CDF method was chosen and maximum likelihood was discarded because its estimates did not fit the observations. (PDF) [file pcbi.1004647.s010.pdf]

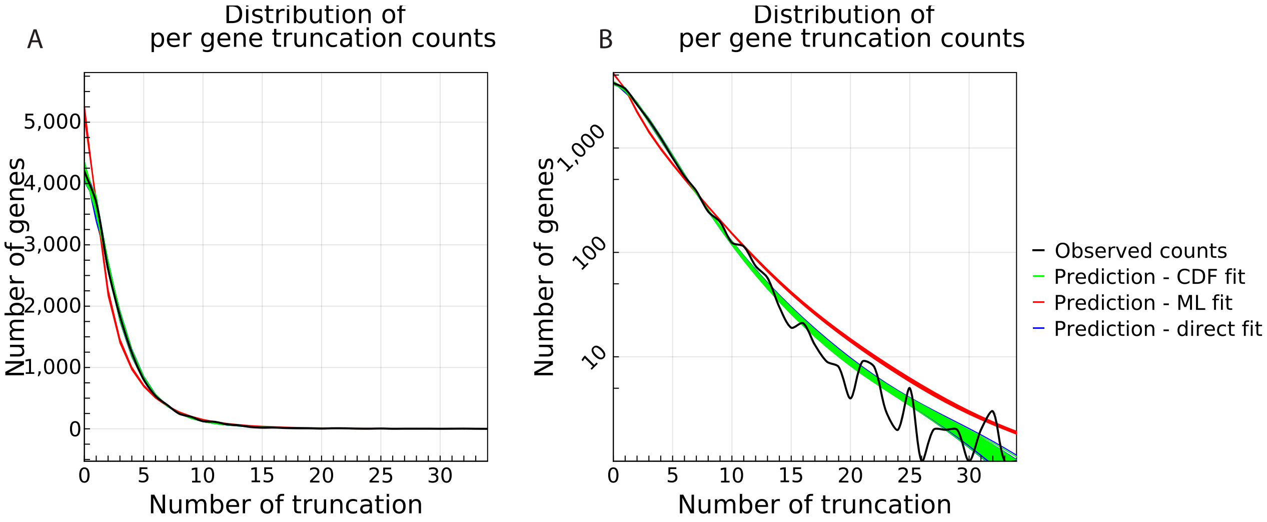

Figure S4: **Distribution of variant counts per gene as observed or predicted under best-fit parameters of model A using 3 different estimation techniques. A:** linear space, **B**: log space. Black curve: observed counts, red curve: prediction based on least-squares fit to the cumulative distribution function (see Online Methods), green curve: maximum likelihood estimate, blue curve: least squares fit to the accumulation curve of truncated genes as shown in Figure 1. The CDF method was chosen and maximum likelihood was discarded because its estimates did not fit the observations.
